# ‘We all have the same right to have health services’: a case study of Namati’s legal empowerment program in Mozambique

**DOI:** 10.1186/s12889-020-09190-7

**Published:** 2020-07-10

**Authors:** Marta Schaaf, Joana Falcao, Ellie Feinglass, Erin Kitchell, Nadja Gomes, Lynn Freedman

**Affiliations:** 1 Brooklyn, USA; 2 Namati Moçambique, Maputo, Mozambique; 3 Namati, Washington, DC, USA; 4 Averting Maternal Death and Disability, Department of Population and Family Health, Mailman School of Public Health, Columbia University, New York, USA

**Keywords:** Legal empowerment, Social accountability, Health systems, Village health committees

## Abstract

**Background:**

Legal empowerment and social accountability are two strategies that are increasingly used to address gaps in healthcare in low- and middle-income countries, including failure to provide services that should be available and poor clinical and interpersonal quality of care. This paper is an explanatory case study of a legal empowerment effort that employs community paralegals and trains Village Health Committees (VHCs) in Mozambique. The research objective was to explore how community paralegals solved cases, the impact paralegals had on health services, and how their work affected the relationship between the community and the health sector at the local level.

**Methods:**

The case study had two components: (1) a retrospective review of 24 cases of patient/community grievances about the health system, and (2) qualitative investigation of the program and program context. The case reviews were accomplished by conducting structured in-depth interviews (IDIs) with those directly involved in the case. The qualitative investigation entailed semi-structured Key Informant Interviews (KIIs) with district, provincial, and national health managers and Namati staff. In addition, focus group discussions (FGDs) were held with Health Advocates and VHC members.

**Results:**

Case resolution conferred a sense of empowerment to clients, brought immediate, concrete improvements in health service quality at the health facilities concerned, and seemingly instigated a virtuous circle of rights-claiming. The program also engendered incipient improvements in relations between clients and the health system. We identified three key mechanisms underlying case resolution, including: bolstered administrative capacity within the health sector, reduced transaction and political costs for health providers, and provider fear of administrative sanction.

**Conclusions:**

This study contributes to the limited literature regarding the mechanisms of legal empowerment case resolution in health systems and the impact of hybrid legal empowerment and social accountability approaches. Future research might assess the sustainability of case resolution; how governance at central, provincial, and district level is affected by similar programs; and to what extent the mix of different cases addressed by legal empowerment influences the success of the program.

## Background

Legal empowerment and social accountability are two strategies that are increasingly used to address gaps in healthcare in low- and middle-income countries, including failure to provide services that should be available and poor clinical and interpersonal quality of care [1–3]. This paper presents an explanatory case study of a legal empowerment program that incorporates strategies of social accountability. The program is being implemented by the NGO Namati in Mozambique.

The field of legal empowerment grew in part out of the recognition that top down efforts to build the capacity of the legal system may be insufficient to effect change in people’s lives. Legal empowerment consists of bottom up efforts to help marginalized people to learn about law and policy, and to use this knowledge to obtain concrete improvements in a relatively short period [2]. Training and deployment of community paralegals or ‘barefoot lawyers’ are one of the most common legal empowerment tactics. Often, paralegals come from the very communities they serve. They function essentially as problem-solvers; they try to bring the remedies enshrined in law and policy closer to communities by working with clients to shepherd complaints through the formal or informal administrative or legal system [3–5].

Legal empowerment organizations increasingly draw on strategies from social accountability to facilitate improvements in service delivery [3, 5]. Social accountability is defined as “the ongoing engagement of *collective* (emphasis added) actors in civil society to hold the state to account for failures to provide public goods” [6]. Common social accountability tactics include organized public dialogues with decision-makers, service providers, and community members; community score card programs; and community monitoring of government services. Social accountability brings collective mobilization to legal empowerment’s concerted focus on the resolution and remedy of individual cases [3, 5].

Legal empowerment and social accountability are apt strategies in Mozambique, which has significant gaps in health service delivery. The continuing HIV/AIDS epidemic, structural adjustment, and other factors undercut investment and progress made in the early 1990s [7]. Patients in the public sector often experience poor quality of care, including provider absenteeism; demands for informal payments for care that is mandated to be free; poorly trained and supervised health providers; frequent drug and supply stock outs; inadequate infrastructure; and disrespectful care [8, 9]. The poor generally lack awareness about their health entitlements, and there are few structured opportunities for patients and communities to provide meaningful feedback or to demand more from the health system [4]. The Ministry of Health has created Village Health Committees (VHCs), which theoretically include representatives of the community. However, these Committees receive little support from the government and generally do not meet regularly, if at all.

Namati Moçambique’s overall objective is to build community capacity to demand quality healthcare and governmental capacity to implement policies to provide care. The program strategy involves: (1) increasing community awareness of health policy, (2) strengthening community involvement in health governance, (3) pursuing solutions to specific breakdowns in health care delivery, and (4) using grassroots data to impact national policy and practice. Namati implements their strategy through a team of paid staff working at multiple levels of the health system. The organization currently has 41 Health Advocates (community paralegals) posted in 19 of Mozambique’s 128 districts. Advocates focus on the first three activities above through several different avenues. First, they conduct health entitlement information sessions in health facility waiting areas and schools, with VHCs, and with grassroots organizations. Each Health Advocate is responsible for one to two health facilities, depending on patient volume and geographic coverage. Health Advocate catchment areas range from 3000 to 120,000 people. Second, Health Advocates train and support VHCs to undertake bi-annual health facility assessments. In some cases, they activate VHCs that had previously been dormant. These assessments, which have been formally recognized by the Ministry of Health, include an objective assessment of facilities, as well as subjective input from health facility staff and the community. Finally, Health Advocates work along VHC members to identify and address individual and collective patient grievances, or cases. Collective cases are defined as issues affecting more than 10 people. Advocates work with the client or the VHC to shepherd these concerns through the administrative system. Table 1 shows the breakdown of case type and resolution rate from March 2013 to September 2018. Table 2 describes typical cases that Health Advocates address. Health Advocates who are unable to resolve cases within a specified period (the timeframe varies according to the type of case) seek assistance from one of the five Namati Program Officers.

**Table 1:** Cases presented to Namati, March 2013 – March 2019

Nature of Case	Status of Case		
	Resolved	In process	Total
Equipment & medical supplies	387	270	657
Infrastructure	751	477	1228
Medicines	302	81	383
Provider behavior	2266	343	2609
TOTAL	3706 (76% of total)	1171	4877

**Table 2:** Types of cases addressed by Health Advocates

Types of cases	Examples
Equipment/medical supplies	• Lack of medical or surgical equipment • Lack of ambulance or fuel for ambulance • Lack of sufficient beds and/or mattresses
Infrastructure	• Lack of electricity /running water • Lack of hygiene at the health facility • Lack of functioning bathrooms for patients • Lack of wheelchair access
Medicines	• Lack of medication • Insufficient dispensing of medicines • Expired medicines
Provider behavior	• Demands for informal payments
	• Provider absence or tardiness
	• Violations of patient confidentiality
	• Disrespectful or abusive treatment
	• Failure to provide information to a patient
	• Refusal to provide services

### Study approach

Namati Moçambique and other legal empowerment programs aim to effect immediate, concrete change through the resolution of cases, and to parlay case related changes into wider improvements in governance at local and national level. The limited research on legal empowerment efforts in the health sector has shown that, when successful, these efforts can increase patient knowledge of entitlements; and engender more respectful treatment of patients, improved health clinic functioning, and improvements in local level health governance [2, 4]. However, little peer-reviewed research has been undertaken on legal empowerment for health overall, and we lack evidence about the mechanisms by which cases are resolved. By mechanisms, we mean the underlying processes operating to produce an outcome [10, 11]. There is a significant body of work on the impacts that participatory development and social accountability can have on governance, including on “state-society relations,” the relationship between communities and the government [12, 13]. However, there has been little exploration of how the resolution of cases typically addressed in health legal empowerment programs might contribute to the wider governance context, including community trust in the government and broader changes in state-society relations. In the context of community health, the local health facility and local officials are key embodiments of the “state” to communities, so changes in the frequency and quality of these interactions might result in broader improvements in mutual trust and cooperation.

### Aims and objectives of the study

Our case study focused on the activities undertaken by the community paralegals (called Health Advocates by Namati), and not on Namati’s entire organizational theory of change. More specifically, the research objective was to explore how Health Advocates solved or did not solve cases, the impact Health Advocate activities had on health services, and how their work affected the relationship between the community and the health sector at the local level. Given the lack of research on how community paralegals affect change, we also sought to identify the mechanisms underlying any changes in health services.

Specifically, we had the following research questions:

- What are the proximal health services impact of Namati Moçambique’s community paralegal program?
- How do Health Advocates solve cases, by what mechanisms are cases resolved, and what enables these mechanisms?
- What is the impact of the program on relationships between communities and the health system?

In exploring these questions, we intended to draw preliminary conclusions about legal empowerment as a means to improve healthcare quality, and to propose avenues for future research.

The research had two components: (1) a retrospective review of 24 cases, and (2) qualitative investigation of the program and program context.

## Methods

In designing this study, we used Yin’s definition of a case study: “an empirical inquiry that investigates a contemporary phenomenon in depth and within its real-world context, especially when the boundaries between the phenomenon and context may not be clearly evident” [14]. This is an explanatory case study, seeking to answer “what,” “how,” and “why” questions. Answering “why” questions is more challenging absent a comparison locality, but comparing the findings from this study to the existing literature enabled us to build deductive, theory relevant study materials, and to undertake some cross-unit analysis [14, 15]. Yin explains that case studies are suitable for studying interventions with no single set of outcomes [14]. An ongoing NGO legal empowerment and social accountability program fits into this category.

Two authors on this paper are employees of Namati. They were involved in study design and manuscript preparation; neither was involved in data collection or analysis. Ethical Approval was obtained the IRB of the Columbia University Medical Center, as well as the National Bioethics Committee of the Mozambican Ministry of Health.

### Site and case selection

The cases and interviewees for the qualitative investigation came from a total of six sites (each site is a health facility) in three districts – Marracuene, Maputo Cidade, and Namaacha. The Namati program started in 2013 in four of the sites, and in 2014 in two of them. Data were collected between April and June, 2016. Nine different Health Advocates worked on the 24 cases selected for study.

At the time the research was conducted, Namati divided their cases into 3 areas of focus – infrastructure, performance, and medicine. The case review entailed the purposeful selection of 24 cases by area of focus with a disproportionate number of performance cases, as these cases had the greatest variation in terms of issues addressed. Namati had a case resolution rate of 88% in the year preceding our study. Given this high rate, we sampled only successful cases for our case review so that we could assess the mechanisms of case resolution. (We asked questions about unresolved cases as part of the qualitative investigation of program context). We sought to achieve maximum variation regarding the route to case resolution. The case reviews were accomplished by conducting structured in-depth interviews (IDIs) with those directly involved in the case: the Health Advocate who managed the case (9 total interviews); the client(s) (22 total interviews); and a representative of the health facility (13 total interviews).

The second component was a broader assessment of the program and the program context, including people’s experience with the program, their feelings on its effectiveness and its impact on broader factors such as trust, and challenges faced. It included semi-structured Key Informant Interviews (KIIs) with district, provincial, and national health managers (5) and Namati staff (4). In addition, focus group discussions (FGDs) were held with Health Advocates (2 FGDs with 7 participants total) and VHC members (6 FGDs with 31 VHC members total).

### Interview and focus group procedures

The IDI, KII, and FGD guides were developed collaboratively by MS, JF, and the Namati team in Mozambique; they were based on Namati’s program theory, as well as the current evidence based for legal empowerment and social accountability. These methods are well-suited to tracing the case process to identify the mechanisms for resolution, and, capturing changes in the perceptions of the health system and in relationships with providers. The IDI guides focused on the trajectory of individual cases, and the way(s) these cases were resolved. To complement this deductive approach, we included a few more open-ended questions to solicit interviewee feedback.

KIIs complemented the case-focused IDIs by allowing us to ask more general questions about experiences with the Health Advocates, how interviewees think cases are solved in general (as opposed to just asking about one case), what they perceive to be the impact of Health Advocates’ work, the challenges Health Advocates face, and the broader context of health systems strengthening in Mozambique. We held FGDs rather than KIIs with VHC members because we expected that the social interaction inherent in FGDs would yield more content as VHC members may have been intimidated or shy in a one-on-one setting. We conducted FGDs with Namati Health Advocates to encourage them to discuss their engagement in the program, how they felt cases were resolved, what the key challenges were, and how the program activities relate to the wider context; we expected that responses to our more general queries would be more fully fleshed out through group discussion.

Two different research assistants conducted the interviews and moderated the FGDs. These assistants had already conducted qualitative interviews in the past, and had familiarity with the Mozambican health system and the broader development context. JF oriented these assistants to legal empowerment and to our research. She accompanied them on their first field visits. The research assistants also pre-tested the interview and FGD guides.

The research assistants spent about a day at the health facility in each field site. They telephoned in advance to receive permission from the health facility, and to set up FGDs with VHC members and appointments with IDI participants. Research assistants asked VHC leaders to gather VHC members who were willing to participate in an FGD at an appointed time at a neutral location (such as under a tree).

Research assistants selected participants for the IDIs regarding case resolution based on their involvement in the case; the community member named in the case (the “client”) and the Health Advocate who handled the case were both interviewed, as well as the relevant health provider or manager. Once present in the health facility, research assistants recruited the district and provincial manager KII participants by asking those present in the health facility to participate; these participants were thus chosen based on their being present at the health facility on the day interviewers visited and their willingness to participate.

Research assistants undertook the KIIs with Ministry of Health representatives and Namati staff and the FGDs with Health Advocates in Maputo. Representatives of the Ministry of Health were invited to participate based on their having had interactions with the Namati program.

All interviews were audio-recorded and conducted in Portuguese. Interviews were transcribed in Portuguese, and then translated into English by a professional translator. JF checked the quality of translations, including back translating a portion of the interviews. Occasionally, clients sought clarification in Changana or preferred to express a particular thought in Changana. These were transcribed in Changana, and translated into English by the Portuguese/Changana to English translator.

### Analysis

Transcripts were saved in Nvivo 11. MS and JF collaboratively developed thematic codes. The initial list of thematic codes was based on relevant studies and Namati’s program theory. The theory reviewed ran the gamut from tentative specific program theories (e.g. [4]) to propositions that developed following an extensive literature review of legal empowerment and social accountability (e.g. [1, 2, 16]). We then modified the codes (including adding some new codes) following review of all the transcripts; thus, the ultimate code list included both inductively and deductively derived codes [17, 18]. Many of the codes related to the trajectory of case resolution; we started with general codes that we then broke down into sub-themes (child codes) as we saw what emerged in the transcripts. For example, we had a code for “mechanism of case resolution;” we then developed more specific child codes reflecting the mechanisms participants had identified. The coding list also reflected questions that emerged from the literature, such as “trust in the health system,” and “issue importance” (e.g. whether the person being interviewed thought the case in question was addressing an important problem). We used codes in a value neutral fashion; we applied them when the phenomena of interest was present, as well as when interviewees explicitly stated that it was not present.

MS coded the transcripts, with substantial input from JF, who discussed the coding of 10 initial transcripts. In addition to coding, we built explanations by developing a data display showing similarities and differences among stakeholder (Health Advocate, client, and provider) perceptions of each of the 24 cases reviewed. To ensure accuracy, two people (MS and a research assistant) assessed all entries in the data display, verifying that we had the same understanding of the transcripts. In addition, we looked for discrepant data in the other data sources to confirm the conclusions that emerged from the data display. The data display facilitated comparison across cases and across type of stakeholder [19–21].

To synthesize the transcripts, MS developed thematic memos including exemplary quotes. The memos explored the range of exemplars for the same codes, illustrating dimensions of the phenomena of interest [20]. To support accuracy (internal validity), MS compiled “quasi statistics” [22] including majority and minority opinions. Comparing the memos with the data display raised just a few areas of seeming disagreement in the data; we then went back to the transcripts to develop nuanced, accurate statements. MS discussed conclusions with other members of the study team. All study team members commented on and contributed to multiple drafts of the paper.

## Results

We begin with a summary of the 24 cases that we included in our retrospective review, the practical steps Health Advocates took to solve the cases, the proximal impacts of case resolution, and the impacts the program had on “state-society relations.” We then describe the mechanisms of case resolution.

The summary of the cases, the practical steps taken to solve them, and the proximal impacts of case resolution were drawn from the data display and from our triangulating among the three IDIs regarding the resolution of each case. The descriptions of the broader program impacts and the description of mechanisms of case resolution were taken from all data sources.

### Cases studied

The cases we retrospectively reviewed are summarized in Table 3. Health Advocates identified the client (person making the complaint) by encountering them at a health facility, through door-to-door visits or education sessions in communities, or through referrals from VHCs. Some of the cases were about problems that affected individual patients; some were about problems that affected many members of the community.

**Table 3:** Description of Namati cases studied

Cate-gory	Case No.	Case Descriptions
Medicine	1	Stock out of a TB drug
	2	Stock out of cotrimoxazole syrup (antibiotic used chiefly as prophylactic treatment for opportunistic infections among HIV+ children)
	3	Stock out of a malaria drug
	4	Stock out of injectable antibiotics
	5	Stock out of a TB drug (expired drugs on site)
	6	Stock out of cotrimoxazole
Infrastructure	7	Lack of appropriate space for TB services
	8	Overflowing clinical waste pit at health facility (staff continued to dispose waste there even though full; terrible smell)
	9	Lack of warehouse for drugs
	10	Facility doors do not have locks, windows do not have screens, broken glass; threat of theft of medicines & lack of security in maternity ward
	11	Toilet broken in men’s ward
Performance	12	Mobile clinic interrupted service for 4+ months w/out explanation to community
	13	Pharmacist told patient seeking ART drugs to come back the next day
	14	Government-employed HIV activist breached confidentiality by disclosing the health status of ART patients to others when drunk
	15	Clinician only observed & gave paracetamol, would not administer malaria test to a sick child
	16	Pharmacist seemingly discriminated against certain patients, not giving them medications; underlying issue was stock out of required drugs.
	17	Clinician told patient seeking wound dressing that facility was too busy & that she should come back at noon
	18	Client not recommended for rapid flow ART even though eligible
	19	Clinician requested bribes for labor and delivery services
	20	Clinician made ART patients wait while allowing other patients (family/friends) to skip the queue
	21	Untrained service agent in maternity attending births b/c nurse on duty did not know how
	22	Patients on hospital beds w/out sheets b/c technician was tired of changing sheets
	23	Mother left alone on a bed w/ no sheets after delivering in the middle of the night
	24	Clinician left a laboring woman alone in the maternity ward; she delivered w/only her mother-in-law & a service agent (not a qualified provider) present, & w/out proper hygiene (gloves)

The problems addressed were manifest at the client’s local health facility, but many of these problems were driven by weaknesses at multiple levels of the health system. For example, drug stock outs at facility level can reflect problems higher up in the supply chain, such as leakage or inappropriate projections at the national level.

### Case resolution

Health Advocates took a variety of steps to resolve these cases. Frequently cited approaches included educating health workers and administrators about how to solve a certain problem and assisting them to do it; facilitating a dialogue among the Health Advocate, the client, and the allegedly offending provider; supporting the process of putting new health facility procedures in place; holding meetings with facility staff; helping the health facility to submit a formal request for funding or support to the district or province; and submitting a letter and/or a petition to the district. Resolving a case often took more than one of these actions. In most cases, the Advocate indicated that at least one VHC member participated in each step.

Table 4 presents three of the 24 cases we reviewed; these are typical, multi-step, multi-level cases:

**Table 4:** Typical processes for resolving cases

Type of Case	Example
**Medicine**	- A Health Committee member reported a stock-out of cotrimoxazole syrup for children - The Health Advocate met with the head nurse, facility director, and head of pharmacy, and was told the facility had a stock of the tablet form of cotrimoxazole and had requested supply of syrup form from the district and province - The facility received a supply of cotrimoxazole syrup from the district - A community member was assigned to oversee the stock at the facility
**Infrastructure**	- A client reported an overflowing clinical waste pit at a facility - The Health Advocate met with the VHC and facility management to discuss the problem, and the health facility management contacted the municipality - When there was no response, the Advocate worked with the VHC to draft and submit a petition to the municipality - The Advocate continued to follow up with the municipality until they sent an excavator to dig a new waste pit - The old waste pit was closed and the VHC began monitoring the facility to ensure waste was being disposed in the new pit
**Performance**	- A client reported that a provider was prioritizing family and friends over other patients awaiting treatment - The Health Advocate and VHC met with the provider - When the behavior continued, the Advocate and VHC met with facility management and recommended escalating the case to district management - The Advocate and VHC drafted and submitted a formal letter to district management - District management met with the provider - The provider was transferred to a different post and reportedly monitored more closely in the new post

Health Advocates and VHC members reported that they continued to directly observe and monitor the situation after a case had been resolved, so that they would know if the problem had re-emerged. They monitored through direct observation (e.g. by visiting a facility), informal interviews with patients exiting the facility, or discussion with the director of a health facility.

### Proximal outcomes of case resolution

According to the interviewees, all of the cases we reviewed had been resolved. For example, stocked out drugs became available, or providers agreed to discontinue certain behaviors and they did so for at least 30 days. In FGDs, VHC members stated that case resolution could have immediate, tangible impact on patients and providers. For example, a functioning toilet that does not stink improves provider working environment and patient experience, and contributes to a more sanitary environment in the health facility. We probed interviewees about whether or not the positive changes resulting from case resolution were sustained. Interviewees reported that to their knowledge, none of the problems recurred, though a few of the resolutions were temporary. For example, the health facility used materials that were on hand to fix an infrastructure problem, while awaiting funds from the next budget cycle to complete permanent repair.

We also found that resolving multiple cases that had a common underlying driver could result in a solution for that driver, though Namati did not track this systematically. For example, facilities with constant challenges due to workload were sometimes allocated an additional health provider. Other facilities obtained needed equipment, such as a CD4 count machine, so that patients no longer complained about needing to travel to a more distant facility for HIV care.

### Empowerment

Clients, Health Advocates, and VHC members stated that that process of learning about their rights and entitlements and resolving a case often brough a sense of empowerment to the client. When asked to reflect on their case, most clients expressed appreciation that they had been able to raise an issue that affected them, and that a Health Advocate and health facility had taken this issue seriously and addressed it.

Interviewees explained that lack of entitlements knowledge, inability to write and thus file a written complaint, the normalization of poor service, and fear of retaliation made community members unlikely to seek redress when their rights or entitlements were not respected. As a Namati staff member explained in a KII, there is a “culture of silence” about rights violations and poor-quality healthcare. However, when they learned about their rights and entitlements and were offered concrete assistance for pursuing redress, many community members were willing to accept assistance from a Health Advocate. Clients describe this individual empowerment:

*“Because before, a patient didn’t know what his rights are, but now he already knows how to demand his rights … I am demanding my rights as a patient, as a user.” (IDI, client)*

*“I'm very happy because I learned a lot with NAMATI, I was a very shy woman, I could not even talk to you, but through NAMATI I can already talk” (IDI, client)*

VHC members who were trained by Health Advocates and who benefited from side-by-side cooperation with Health Advocates widely agreed that they, too, felt more empowered. As described by one VHC member:

*“The health committee became more motivated by NAMATI’s presence, because NAMATI gave us experiences that we didn’t have before, so we became more composed with the experience of NAMATI, we have more strength, more will because of NAMATI” (FGD, VHC)*

Health Advocates, VHC members, and Namati staff explained that entitlements education and the successful resolution of a case helped to ‘de-normalize’ poor quality services, thus instigating a virtuous cycle of community engagement and willingness to pursue more cases. As a client and VHC member explains:

“*Once the Health Advocate had solved a few cases, the community began to express thanks and had more courage to come to present more problems.” (IDI, client who is also a VHC member)*

We explicitly asked interviewees about who was being empowered, and whether and how the most marginalized were benefiting from Health Advocates’ work. We did not find any evidence that the benefits were accruing disproportionately to those who already had more power in the community, such as community leaders and men (as opposed to women). For example, one VHC member explained:

*“[Namati] advises us so that there is no discrimination in the hospital, so that they meet all patients well” (FGD, VHC)*

Health Advocates also explained that they supported clients who – for whatever reason – were treated less well in their local health facility, such as women who were asked to labor on the floor, and that they conducted outreach with marginalized groups in particular, including the elderly, people with TB, people with HIV, and orphans and vulnerable children.

Among other outcomes, Namati intended for patient empowerment to enhance client comfort in the health facility. A VHC member explained this objective:

*“Patients should feel like they are at home when they are in the hospital, to express themselves freely about their health concerns.” (FGD, VHC members)*

Many of the cases addressed factors that shaped client comfort in the health facility; their resolution made patients feel more respected and/or that the facility was more transparent and fair in its operations. For example, with Health Advocate support, VHC members worked to triage and organize the queue at health facilities. As a VHC member explained:

*“Those cases can’t happen that this one [patient] is [a health provider’s] neighbor and he should be the first to be attended to, because we all have the same right to have health services.” (FGD, VHC members)*

*Interviewee: I will always be in the queue because I don’t know someone, you see …*

*Interviewer: Ok, so today you see that the queue is well organized, you think it was because of you having complained?*

*Interviewee: Yes. (IDI, client)*

One interviewee noted that some successful Namati cases regarding informal payments were raised by patients who had been skipped in the queue because they were unwilling to pay. These clients brought their concerns to Health Advocates because they wanted a more affordable, transparent, and/or democratic process for receiving care.

However, our data also indicated that Health Advocates did not always “close the loop” with clients, undercutting client empowerment. Health Advocates did not tell them about the outcome of their particular case and educate them more broadly about the potential impact of rights claiming. Thus, though their cases were resolved, of the clients interviewed for the case review, almost half indicated that they wished they had more follow up from the Health Advocate.

Through ongoing monitoring and evaluation, Health Advocates had become aware of this weakness in the feedback loop. One Advocate describes the impact of inadequate follow-up:

*When the burglar bars were put at the health facility and the other windows were repaired … there was no demonstration to the patients that this is the result of the complaints, of empowerment on the right to health (IDI, Health Advocate)*

Namati has since taken steps to address this gap.

### Mechanisms for case resolution

We identified three key mechanisms for case resolution: (1) increased administrative capacity within the health sector, (2) reduced transaction and political costs for health providers and officials, and (3) sanctions or fear of sanction among health providers.

In many situations, Health Advocates directly bolstered health facility level (and sometimes, district level or municipality) administrative capacity to address and prevent the problems identified. Health Advocates used several strategies to improve administrative capacity. Interviewees explained that Health Advocates worked with health facility providers and administrators to learn about new protocols or about lesser-used processes for fixing problems, such as referring adherent HIV patients to a rapid flow for anti-retroviral treatment or asking the municipality for funds for infrastructure repairs. Health Advocates explained that their experiences in addressing similar cases in multiple health facilities enhanced their ability to identify and remedy problems. They also described the broader context: health care planning and management was being decentralized from the provincial to the district level, human resources were inadequate, and HIV treatment scale-up put significant burdens on health provision. Over-worked providers and managers understandably had trouble mastering administrative procedures and fast-changing protocols. Health Advocates learned about some of these procedures and protocols, and taught both VHC members and relevant health facility employees about them. In some cases, they worked with health facility staff to implement them, such as working with them to draft letters to request funding from district authorities. In addition, in some cases, Advocates worked with VHCs to create new management systems, thus more sustainably improving capacity. For example, several VHC members reported proactively reaching out to the pharmacist every 15 days to check on stock levels of key medicines.

This mechanism was enabled in part by the fact that Health Advocates worked to establish trusting relationships with health providers, and the fact that providers and administrators often welcomed these capacity improvements. Health Advocates and VHCs addressed many problems health managers and providers felt were important. Although a minority of health providers and officials opined that they would prefer that Namati work only on educating community members about how to take better care of their health and not on rights and entitlements education and realization, these sentiments were outweighed by more widespread appreciation of Health Advocates’ work.

When asked directly during the case review interviews, most providers agreed that the case under discussion did indeed relate to an important problem. Providers unequivocally stated that drug stock outs and infrastructure gaps in particular undercut their work as medical providers. When discussing a Health Advocate effort to improve infection control by creating a segregated area for TB patients, a doctor explained:

*“For me as a clinician, we knew we were exposing the providers and patients to a range of diseases… It [resolving this case] was a great victory.” (IDI, health provider)*

As shown, health providers accepted assistance in part because they wanted to fix the problems identified. Our data also suggested that health facility staff accepted capacity assistance because they had grown to trust Health Advocates. Health facility staff may have initially questioned Advocate motives, but this circumspection gave way to a constructive working relationship once the providers understood that they shared some goals with the Health Advocate. A Health Advocate described this evolution:

*I can say that when I started working as an Advocate, it is always like that, when you start working at a health facility, you are frowned upon, you are frowned upon by the providers, only when they understand what the objective is, what the role of the Advocate is, that is when they get closer to you (IDI, Health Advocate)*

When asked about Namati, providers described Health Advocates as acting in good faith. Resolving some cases required uncomfortable interactions, but the working relationships between Advocates and providers seemed to withstand this discomfort. Advocates indicated that Namati training and support helped them to build these functional relationships with providers. Namati trained Health Advocates used an overarching framework of entitlements and right to health care, but they also taught Advocates to approach health providers as allies and fellow professionals with shared goals. A Health Advocate described this approach:

*Interviewee: Namati taught me how to follow up, how to deal with the nurses, how to speak like that.*

*Interviewer: Like that, how?*

*Interviewee: Knowing how to connect with the nurse, have manners, good language, not to judge, not to police the nurse, knowing how to connect with them (Health Advocate, IDI)*

Another Advocate expressed similar sentiments; this quote too, is emblematic.

*I will start as I was taught in Namati, I began to follow the steps and then I saw that after all it is not that hard … not to go in there and act as if you were police, or go in to judge, no, you go in as if you were working with them, seek to know what it is like there (Health Advocate, IDI).*

Health Advocates’ accompaniment of frontline providers and managers through administrative processes enabled the second mechanism we identified: decreased transaction and political costs of addressing problems. Health Advocates engagement in resolving problems took some of the onus off providers, who for reasons related to professional norms of hierarchy were unable to solve these problems themselves. Pointing out uncomfortable truths about colleagues – particularly about one’s superior - may be politically costly. As outsiders, Health Advocates can more safely raise these issues. A Health Advocate recounted how a health provider described a colleague who was the subject of a Namati case:

*"It’s true that my colleague … really needs to be called to account, but we can’t do this, we are his direct colleagues, but you can do it, you are the community … you can." (IDI, Health Advocate)*

In fact, one of the cases we reviewed was brought to the attention of a Health Advocate by a provider; when interviewed, Namati staff and Advocates confirmed that providers ask Health Advocates for help with some regularity.

*“A lot of times the health providers have said that [prior to alerting the Health Advocate] they made the request [to address a problem internally] … and they give up because they don’t want to pressure their supervisors.” (IDI, Namati staff)*

Our data suggested that administrative accompaniment and decreased political and transaction costs may be the most salient determinants of state responsiveness to Namati Moçambique, as interviewees of all types described case resolution processes that were solved via these mechanisms most frequently during the case review discussions.

In addition, it is also clear that the mechanism of sanction (actual sanctions or fear of them) played a role in some cases. Cases were resolved because health managers and providers feared punishment or did face some kind of sanction. District and provincial officials confirmed that problems are solved by “calling attention” to them, such that the individuals responsible were embarrassed and agreed to take the necessary steps to resolve the problem identified. Officials also noted that egregious cases of poor performance raised by Health Advocates have led to disciplinary measures against providers. We did not ask directly about sanctions during the interviews, but several interviewees raised the issue of sanctions and the need for them themselves:

*“The Advocates … can help us in this fight because nurses work without supervision and they do what they please.” (FGD, VHC member)*

Another Advocate explained that lack of accountability has becomes routine among some providers. The government:

*“employ [s] someone without supervising the person; the person gets accustomed to [lack of supervision] and thinks that this is his [right].” (IDI, Health Advocate)*

Empowered community members and heightened likelihood of attention and sanctions for poor performance created some fear among providers. One VHC member explained that a case served as:

*“a reminder and a warning … we had seen this problem and … they [health providers] would run the risk of being expelled because the community is getting empowered; [the community] are able to take a case until the district headquarters.” (FGD, VHC member)*

## Discussion

Our findings illustrate the types of cases Health Advocates solve, the steps that Health Advocates and VHC members typically take to resolve cases, the proximal impact of case resolution, and the underlying mechanisms that were at play. Proximal impacts include improvements in service delivery, individual empowerment, and incipient changes in state-society relations at the local level.

These findings regarding individual empowerment are ubiquitous in the legal empowerment and social accountability literature; individuals who are not punished for raising a concern and/or who see benefit from an effort start to feel as if they have the right and capacity to speak and ‘make a difference’ [23, 24]. Studies of social accountability efforts have identified two key threats to empowerment - backlash from agents of the state [1, 25, 26] and “elite capture” [16]. We did not find evidence of these occurring. First, interviewees did not describe backlash from health providers or officials. Generally speaking, while Namati was working closely with VHCs, they did not seek to mobilize collectivities within the community at large. It is possible that not fostering collective engagement and Namati’s focus on building trust with facility providers facilitated Health Advocates’ entrée into health facilities, where they were received as problem-solvers rather than as external agitators.

“Elite capture” refers to the domination of participatory processes by individuals in the community who already had more power [16]. Community dialogues and other social accountability approaches may result in action plans that reflect the priorities of the individuals in communities who have the greatest ability to speak up, such as community leaders and men. In contrast, legal empowerment focuses on those who experience a violation. The impact – such as the Namati queue jumping example – may be to make the service delivery process more transparent and impartial for those who are less likely to benefit from community participation approaches.

Frontline service delivery improvements such as those we identified (e.g. repairs, better implementation of policy) are a common outcome in successful legal empowerment and social accountability efforts [1]. In the context of the much-lamented gap between health policy and practice, performance improvements at the frontlines of the health system can be significant to both patients and providers [27]. However, based on the peer-reviewed and grey literature, it seems that the Namati legal empowerment project tracks these outcomes more closely than most social accountability projects. The case flow protocol stipulates timeframes for resolution and when Health Advocates should engage senior Namati employees, and a case management database tracks each step Health Advocates and others take. This reinforces organizational focus on case resolution, and supports organizational learning. This tracking of processes and outcomes already informs Namati’s contributions to national level discussions in Mozambique; it could also be a useful contributor to theory building around successful approaches to problems that occur in many countries, such as demands for informal payments.

Our study found that Health Advocates and VHC members fostered improved, more transparent interactions between health facilities and the communities they serve, improving the micro-level of state/society relations. Patients knew better how to navigate the health system and what their entitlements were. These are forms of capital that patients could leverage in the future. While there is ample literature on social accountability efforts fostering trust between communities and local health facilities, the legal accountability literature generally does not explicitly engage questions about relationships between communities and the government, other than through the lens of empowerment [28].

A common approach in health is to look at the “supply side” (health providers and facilities) and the “demand side” (patients and communities). Here, we are concerned with the interface between the two. Assessing local level changes in state-society relations may be key to understanding the potential of legal empowerment approaches, as strengthened relationships between the health system and citizens can shape the sustainability and transformative potential of the immediate improvements in service coverage and quality that result from resolving individual complaints. Lack of power and access to decision-making structures is a defining feature of marginalization, making it quite difficult for the most marginalized to express voice and demand accountability [29]. The impact of this lack of power is evident in health, as demonstrated by studies showing that in poorly governed health systems, low quality care is often meted out to “anonymous users” who have no relationships or specialized knowledge on which to draw in negotiating health care [30]. Combining relationship building with procedural or legally-based institutional accountability in the way that legal empowerment does may help to ensure that this relationship building works in the interest of the poorest [31].

On the other hand, legal empowerment is focused on individual cases. While the resolution of those cases may benefit the community as a whole, changes in empowerment and trust may occur only among those who directly participate in the program, i.e. the named client in a given case. The Health Advocates engaged the VHCs, extending program activities to a group that is formally mandated to represent their communities, so it may be that through VHCs, the program has started to impel deeper changes in state-society relations at the local level. Exploration of how and under what conditions legal empowerment contributes to community level changes in state-society relations is an important area for further research.

The Namati program (and legal empowerment more generally) also merits examination for the way it affects the knotty challenge of state capacity. Numerous empirical analyses identify state capacity to respond to social accountability efforts as a central concern [16, 29]. Demanding more from the state may be futile and demoralizing in environments where public sector employees and bureaucrats lack the technical capacity, resources, desire, or incentive structure to respond to those demands. Though social accountability programs usually rely on national level standards as a benchmark, these programs typically operate with a bottom up logic. Collectives at the community level seek to effect change at the frontlines of the health system. Desired change occurs when the collectives generate adequate countervailing power, and the frontline health providers and managers have the decision space and resources to respond to these demands [16]. The legal empowerment approach is different. Namati Moçambique’s concerted focus on case resolution can bring enhanced focus to state response at the local level, and their bolstering of facility, district, or municipal administrative know-how can facilitate the state’s response. In this way, the project starts to address this challenge of inadequate decision space and resources. These mechanisms of change are not prominent in the legal empowerment or the social accountability literature, though the public sector performance impact of poor state administrative capacity at the local level is well documented [32]. In addition to addressing administrative capacity gaps, Health Advocates’ accompaniment of frontline providers and managers through administrative processes appeared to decrease the transaction and political costs of solving problems, a finding that has been identified in some studies of social accountability projects [33, 34].

In many cases, Health Advocates and VHCs addressed problems health managers and providers felt were important, facilitating their success. This congruence in interests is not minor; erroneous assumptions about shared priorities between the public sector and citizens have plagued many development efforts [35]. This success in addressing shared priorities raises questions about the mix of cases that Health Advocates address. It is possible that Health Advocates’ solving cases providers and managers felt were important built a stock of good or will or reputation that Health Advocates were able to leverage for the cases where their role was less welcomed. While the relevance of type of problem to be addressed has been explored in discussions on politics and accountability [36], we were unable to find a discussion of legal empowerment case mix in the literature. This issue is ripe for further exploration; to what extent do community paralegals build up good will when they address shared priorities and, what happens when they end up with relatively more cases that decision-makers do not feel are important?

This explanatory case study is an early step in building a broader literature on legal empowerment for health. As such, it has several limitations. First, our research was cross disciplinary and included multiple research foci. This required background reading, tools, and a manuscript that address multiple audiences and fields, including legal empowerment, social accountability, health and human rights, and health systems researchers. We did not assume that interviewees or the readers of this paper were familiar with all of those fields, so we spent time on explanation and interpretation. As a result, we were unable to go into as much depth as we wanted to address specific questions that are important in specific fields, such as the long-term durability and impact of empowerment, the added value of a joint legal empowerment and social accountability approach, and health systems responsiveness. Second, this study focuses on one aspect of the Namati Mocambique program – community paralegals (Health Advocates). Since we began the research, Namati has further expanded their work with VHCs and with health authorities. Studying the full universe of Namati activities would shed more light on the impact of this approach to legal empowerment, and potentially on the contextual factors that shape the impact of community paralegals. Third, many qualitative studies are vulnerable to social desirability bias; studies of NGO-run projects may be especially vulnerable. Interviewees of all types may want to tell interviewers what they think the interviewers want to hear, and/or they may want to help the NGO and its staff by describing the program as successful. We tried to overcome this potential bias by triangulating carefully among interviewees, and by seeking out discrepant data. Observation or other non-interview methods may help to mitigate social desirability bias in future research.

## Conclusion

Though they are unable to address some deeply embedded national challenges, such as lack of adequate human resources in the health system, Namati Moçambique’s Health Advocates successfully solved a variety of cases affecting poor Mozambicans.

Health Advocates resolved visible problems that community members themselves had identified, leading to individual client empowerment, and to some extent, prompting a virtuous circle of rights claiming. In resolving these cases, Namati bolstered individual and health facility capacity to provide quality services, and relieved some of the transaction and political costs that may prevent health providers or administrators from addressing the problems by themselves. Case resolution was aided by by the fact that Health Advocates established working relationships with health providers and managers and addressed many challenges that health providers agreed were priorities.

This study contributes to the limited literature regarding the mechanisms of legal empowerment case resolution in health systems and the impact of hybrid legal empowerment and social accountability approaches. Future research might assess the sustainability of case resolution; how governance at central, provincial, and district level is affected by similar programs; and to what extent the mix of different cases addressed by legal empowerment influences the success of the program.

## Data Availability

The datasets generated and analyzed during the current research are not publicly available as individual privacy could be compromised, but they are available from the corresponding author on reasonable request.

## Abbreviations

FGD: Focus group discussion
IDI: In-depth interview
KII: Key informant interview
LMICs: Low- and middle-income countries
MDG: Millennium Development Goal
TB: Tuberculosis
VHC: Village Health Committee
